# Prenatal Diagnosis, Course and Outcome of Patients with Truncus Arteriosus Communis

**DOI:** 10.3390/jcm13154465

**Published:** 2024-07-30

**Authors:** Aline Wolter, Annika Haessig, Andrii Kurkevych, Jan Weichert, Stephan Bosselmann, Gunther Mielke, Ivonne Alexandra Bedei, Johanna Schenk, Ellydda Widriani, Roland Axt-Fliedner

**Affiliations:** 1 Division of Prenatal Medicine, Department of Obstetrics and Gynecology, Justus-Liebig University and University Hospital UKGM Giessen, 35392 Giessen, Germany; annika.r.haessig@med.uni-giessen.de (A.H.); ivonne.bedei@gyn.med.uni-giessen.de (I.A.B.); johanna.schenk@gyn.med.uni-giessen.de (J.S.); ellydda@hotmail.de (E.W.); roland.axt-fliedner@gyn.med.uni-giessen.de (R.A.-F.); 2 Pediatric Heart Centre, 04050 Kiew, Ukraine; akurkevych@gmail.com; 3 Division of Prenatal Medicine, Department of Obstetrics and Gynecology, University Hospital of Schleswig-Holstein, Campus Lübeck, 23538 Lübeck, Germany; 4 Prenatal Medicine, Prenatal Care Center Stuttgart, 70184 Stuttgart, Germanycgmielke@t-online.de (G.M.)

**Keywords:** truncus arteriosus communis, prenatal diagnosis, prenatal outcome prediction, congenital heart disease

## Abstract

**Background**: The objective of our study was to assess the prenatal course, associated anomalies and postnatal outcome and the predictive value of various prenatal parameters for survival in prenatally diagnosed cases of truncus arteriosus communis (TAC). **Methods**: We evaluated cases from four centers between 2008 and 2021. **Results**: In 37/47 cases (78.7%), classification into a Van Praagh sbtype was possible, most had TAC type A1 (18/37 = 48.6%). In 33/47 (70.2%) with available valve details on common trunk valve, most presented with tricuspid valves (13/33 = 39.4%). In the overall sample, 14/47 (29.8%) had relevant insufficiency, and 8/47 (17%) had stenosis. In total, 37/47 (78.7%) underwent karyotyping, with 15/37 (40.5%) showing abnormal results, mainly 22q11.2 microdeletion (9/37 = 24.3%). Overall, 17/47 (36.2%) had additional extracardiac anomalies (17/47 = 36.2%). Additional intracardiac anomalies were present in 30/47 (63.8%), or 32/47 (68.1%) if coronary anomalies were included. Four (8.5%) had major defects. Two (4.3%) intrauterine deaths occurred, in 10 (21.3%) cases, the parents opted for termination, predominantly in non-isolated cases (8/10 = 80.0%). A total of 35/47 (74.5%) were born alive at 39 (35–41) weeks. Three (8.6%) pre-surgical deaths occurred in non-isolated cases. In 32/35 (91.4%), correction surgery was performed. The postoperative survival rate was 84.4% (27/32) over a median follow-up of 51.5 months. Initial intervention was performed 16 (1–71) days postpartum, and 22/32 (68.8%) required re-intervention. Regarding prenatal outcome-predicting parameters, no significant differences were identified between the survivor and non-survivor groups. **Conclusions**: There exist limited outcome data for TAC. To our knowledge, this is the largest multicenter, prenatal cohort with an intention-to-treat survival rate of almost 85%.

## 1. Introduction

Truncus arteriosus communis (TAC) is a rare congenital heart defect characterized by the absence of septation of the embryologic conotruncus [1]. The incidence of this rare cardiac defect is approximately 1.07 per 10,000 live births [2]. The defining feature of TAC is the presence of a single, common arterial trunk, which gives rise to the coronary arteries, the pulmonary arteries and the systemic arterial circulation. In the majority of cases, the arterial trunk overrides a large ventricular septal defect (VSD). The single semilunar valve may contain two, three, four or even more leaflets and may also be dysplastic [3]. When the aortic isthmus is well developed, the ductus Botalli is typically absent or poorly developed, and vice versa [4]. Very rarely, there is no VSD; in such cases, it presents with separately formed but conjoined aortic and pulmonary semilunar valves [3].

**Figure 1:**
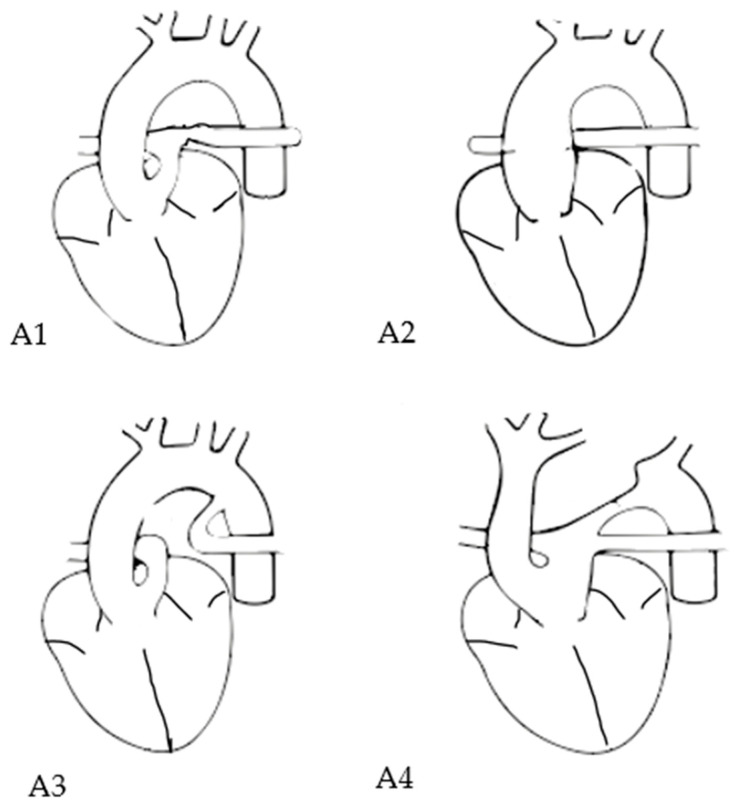
Van Praagh subtypes in TAC. Subtype A1: main pulmonary artery is present and bifurcates into the left and right pulmonary arteries; subtype A2: right and left branch pulmonary arteries arising from a common trunk; subtpe A3: one pulmonary artery branch arising from the common trunk and the other branch from the aorta; subtype A4: presence of aortic arch hypoplasia, coarctation or interrupted arch.

Two principal classification systems exist, with some overlap: the Collet and Edwards classification system and the classification system proposed by Van Praagh. In addition to the aforementioned classification, Van Praagh proposed an anatomical classification system that considers additional aortic arch anomalies and the presence or absence of a VSD [4,5]. The different subtypes of Van Praagh classification are presented in Table 1 and Figure 1.

Additional cardiac anomalies associated with TAC include right aortic arch, persistent left superior vena cava, aberrant right subclavian artery and atrial septal defect [3].

The prevalence of associated extracardiac and genetic anomalies in patients with TAC is approximately 37%. Particularly common associated anomalies include gastrointestinal malformations and 22q11.2 microdeletion, occurring in 25% of cases, as well as trisomy 21 and Turner syndrome [6].

Differential diagnosis of conotruncal anomalies may be challenging particularly in the case of TAC and pulmonary atresia with ventricular septal defect (PA-VSD) during the fetal period. In both cardiac defects, a single arterial trunk overriding a VSD can be identified. Differentiation is based on the characteristics of the arterial trunk valve, detection of an atretic pulmonary valve and the presence or absence of a ductus arteriosus [7].

In the postnatal period, symptoms vary depending on the presence of a ductus arteriosus Botalli, the amount of the pulmonary blood flow and the degree of truncal valve insufficiency [8]. TAC is a cyanotic cardiac defect characterized by increased pulmonary blood flow after birth, caused by a decline in pulmonary resistance. If left untreated, this can lead to progressive heart failure [4].

Data regarding outcome of prenatally diagnosed cases are limited and based on small cohorts [7,8,9,10,11,12,13,14,15]. Prenatal diagnosis is of considerable importance for several reasons. Firstly, TAC is associated with chromosomal anomalies, particularly 22q11.2 microdeletion. Secondly, prenatal diagnosis allows the identification of further cardiac and extracardiac anomalies. Surgical repair of TAC in the early neonatal period has become the standard procedure [3,8,16]. The complete surgical repair procedure comprises closure of the VSD, a conduit from the right ventricle to the pulmonary artery and, in the case of type 4, the reconstruction of the left ventricular outflow tract. During childhood, re-interventions, such as dilatation or exchange of the RV–PA conduit, are frequently required due to recurrent stenoses or insufficiencies [8].

The primary objective of our study was to assess the prenatal course, associated anomalies and postnatal outcome in prenatally diagnosed cases of TAC. Additionally, the objective was to assess the predictive value of various prenatal parameters for survival during the follow-up (FU) period.

## 2. Materials and Methods

This is a retrospective study utilizing a database of newborns with prenatal diagnosis of TAC and postnatal ascertainment at four tertiary centers, between 2008 and 2021: Division of Prenatal Medicine, Department of Obstetrics and Gynecology, Justus-Liebig University and University Hospital UKGM Giessen, Germany; Pediatric Heart Centre, Kiew, Ukraine; University Hospital of Schleswig-Holstein, Campus Lübeck, Germany and Prenatal Care Center Stuttgart, Germany. Fetal echocardiography was conducted in accordance with the international guidelines of ISUOG, employing a segmental approach and defining anatomical planes with color Doppler and pulsed-wave interrogation [17]. Cardiovascular analysis was performed using two-dimensional, color and pulsed-wave Doppler echocardiography, followed by postnatal echocardiography conducted by a pediatric cardiologist after birth.

**Figure 2:**
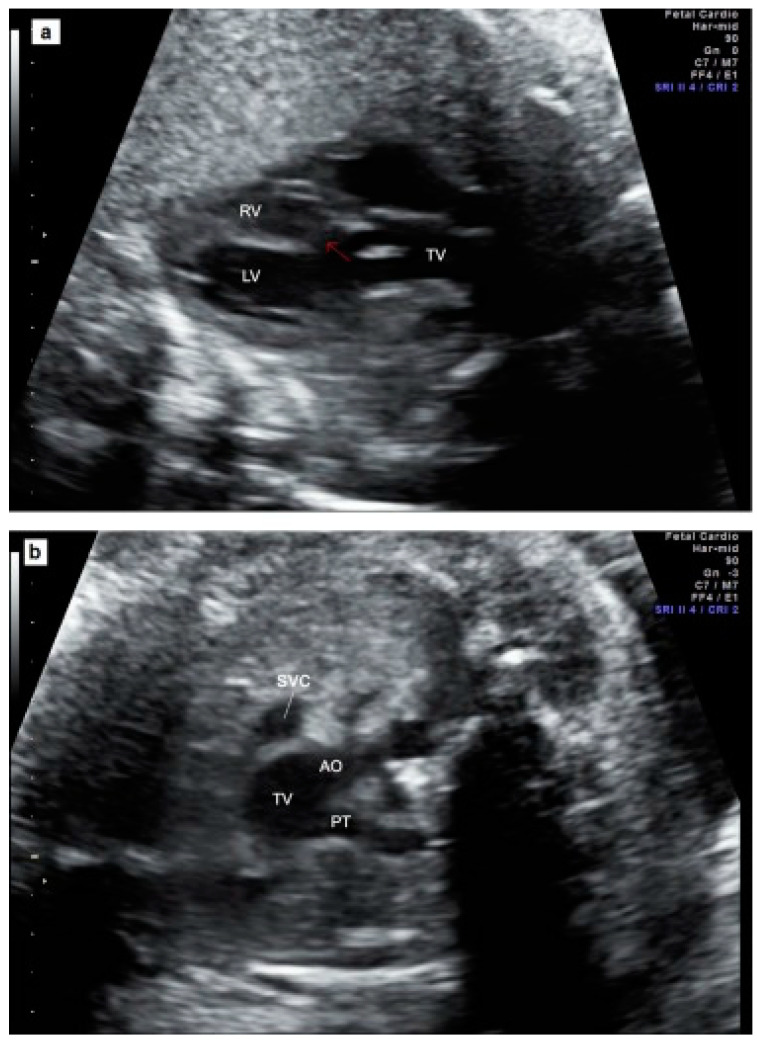

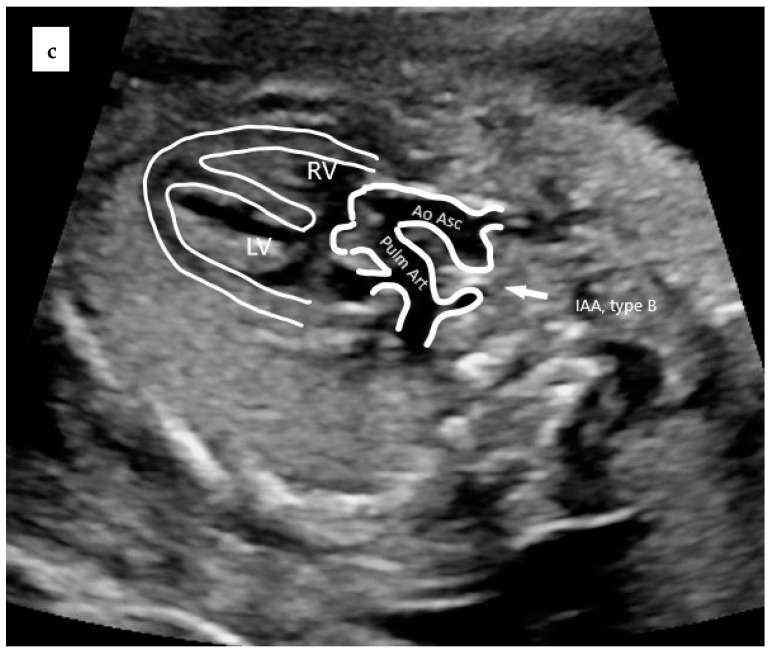
Prenatal echocardiography of TAC. (**a**) The five-chamber view showing the presence of a thickened truncal valve that overrides a large VSD (arrow); (**b**) The three vessels view showing two vessels: superior vena cava and truncal vessel (subtype A1): pulmonary trunk arising from the truncal vessel); (**c**) example of subtype A4 in which aorta and MPA arise from the common arterial trunk and interrupted aorta divides into brachiocephalic and left common carotid arteries (IAA Type B). TV = truncal vessel; VSD = ventricular septal defect; LV = left ventricle; RV = right ventricle; PT = pulmonary artery trunk, Ao Asc = ascending aorta; Pulm Art = pulmonary artery; IAA = interrupted aortic arch.

Figure 2 illustrates an example of a prenatal case with TAC. Parental counseling by pediatric cardiologists and geneticists was part of the prenatal work-up. Karyotyping including fluorescent in situ hybridization for 22q11.2 microdeletion was offered. The data were collected from medical files, as well as from stored ultrasound images and video loops, whenever they were available. 

The parents provided informed consent for the anonymized analysis of the data, and the study was approved by the local research ethics committee (protocol number 31/16).

The statistical data analysis was conducted using Excel (Microsoft Office 2021). Continuous variables are presented as either the mean ± SD or the median (range), depending on the distribution of the data. Categorical data are expressed as frequencies (n) and percentages (%). In order to evaluate the predictive capacity for outcomes, the following parameters were compared between the group of survivors and the group of non-survivors (defined as intrauterine death or postnatal death) during the follow-up period: TAC subtype, the presence of additional anomalies (e.g., chromosomal, extracardiac or intracardiac anomalies; fetal growth restriction (FGR)) and echocardiographic parameters (e.g., the number of valve leaflets; truncal valve stenosis or insufficiency). A chi-square test was employed for the purpose of comparing the two outcome groups.

## 3. Results

A total of 47 cases with a prenatal diagnosis and postnatal confirmation of TAC from four different centers were included in the analysis. The median maternal age was 30 (17–39) years, and the median gestational age at diagnosis was 22 (12–37) weeks. In 37/47 (78.7%) cases, prenatal classification into a Van Praagh subtype was possible, and it was exact in 37/37 (100%). A plurality of patients had TAC type A1 (18/37 = 48.6%), followed by TAC type A2 (13/37 = 35.1%). In 33/47 (70.2%) cases, data regarding valve details on common trunk valve were available. The majority of these cases presented with tricuspid valves (13/33 = 39.4%) and quadricuspid valves (12/33 = 36.4%). Fourteen of forty-seven (29.8%) had relevant insufficiency of truncus valve, and eight (17%) had valve stenosis. The data pertaining to TAC subtype and valve details are summarized in Table 2. 

### 3.1. Additional Anomalies

**Figure 3:**
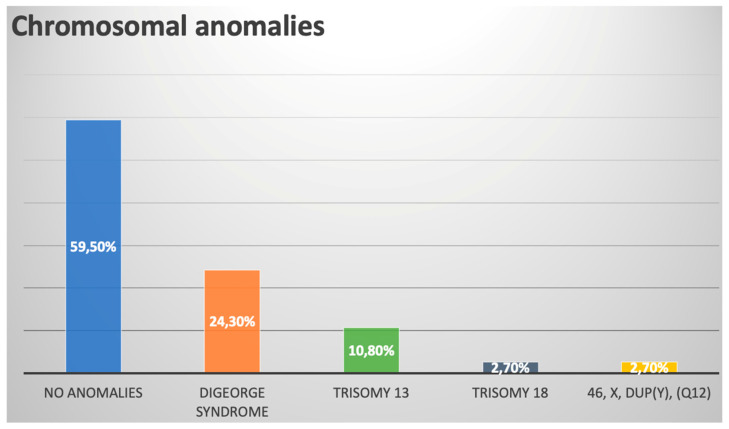
Chromosomal anomalies within our prenatal TAC cohort.

In total, 37 of 47 (78.7%) patients underwent karyotyping, and 15/37 (40.5%) had abnormal results. Nine of thirty-seven (24.3%) patients had 22q11.2 microdeletion, which constituted the most prevalent genetic anomaly within the cohort (9/15 = 60.0%). A summary of chromosomal anomalies is provided in Figure 3. 

Over one-third of patients had additional extracardiac anomalies (17/47 = 36.2%). These anomalies were frequently associated with chromosomal anomalies (6/17 = 35.3%). Seven patients had (14.9%) fetal growth restriction (FGR). 

A summary of the associated extracardiac malformations is provided in Table 3. 

An additional intracardiac anomaly was present in 30/47 (63.8%), excluding coronary anomalies. When coronary anomalies were included, the rate was 32/47 (68.1%). Four (8.5%) patients had major cardiac defects, including hypoplastic heart, atrioventricular septal defect (AVSD) and multiple ventricular septal defects (VSD). The majority of cases had minor cardiac defects, including atrial septal defect (ASD) in 19/47 (40.4%), right aortic arch (RAA) in 13/47 (27.7%) and persistent left superior vena cava (LPSVC) in 4/47 (8.5%) of cases. In eight (17.0%) patients, anomalies of the coronary arteries were observed, including high take-off of the right or left coronary artery (/LCA/RCA), singular coronary artery (SCA), intramural LCA or RCA or abnormal origin of LCA. 

Table 3 (with extracardiac anomalies) and Table 4 (without extracardiac anomalies) provide an overview of the details of associated intracardiac malformations.

Four (8.5%) patients had chromosomal anomalies without any additional extracardiac or intracardiac anomalies (Table 5). 

In seven (14.9%) cases, TAC was isolated without any additional cardiac (excluding anomalies of the coronary arteries) extracardiac or chromosomal finding. Including anomalies of the coronary arteries, only 6/47 (12.8%) cases were truly isolated. (Table 6). 

### 3.2. Outcome

A total of 35 out of the 47 cases (74.5%) resulted in live births at 39 weeks of gestation (ranging from 35 to 41 weeks), with a median birth weight of 3000 g. The mode of delivery was known in 30/35 (85.7%) cases. The majority of patients, 20/35 (66.7%), were delivered vaginally, with 18/20 (90.0%) being spontaneous deliveries and 2/20 (10.0%) being operative vaginal deliveries. 

With regard to outcomes during the prenatal period, two (4.3%) cases of intrauterine death (IUD) were documented. One of these cases involved additional extracardiac anomalies (single umbilical artery and agenesis of ductus venosus) and occurred at 29 + 0 wks. The other case was isolated and also occurred at 29 + 0 wks of gestation. In ten (21.3%) cases, the parents opted for termination of pregnancy, with the majority (8/10 = 80.0%) representing non-isolated cases. 

A total of 35 out of the 47 (74.5%) cases resulted in live births at 39 (35–41) wks of gestation with a median birth weight of 3000 g. The mode of delivery was known in 30/35 (85.7%) cases. The majority of patients, 20/35 (66.7%), were delivered vaginally, with 18/20 (90.0%) being spontaneous deliveries and 2/20 (10.0%) operative vaginal deliveries. 

Three (8.6%) patients died prior to surgery. Two of these deaths were due to multiorgan failure following sepsis, while the third occurred two hours postpartum due to cardiac and pulmonary failure. The three cases in which death occurred prior to intervention were non-isolated cases associated with relevant extracardiac anomalies (esophageal atresia, biliary atresia and renal hypoplasia + SUA, hydronephrosis, renal agenesis, hemivertebra, plantar cutaneous appendage). One of these cases also involved a 22q11.2 microdeletion and prematurity at 35 wks. 

Correction surgery was performed in 32/35 (91.4%) of cases. The postoperative survival rate was 84.4% (27/32), with a median postnatal FU period of 51.5 (9–147) months.

Initial intervention was performed 16 (1–71) days postpartum in median. A total of 22 out of the 32 (68.8%) required re-intervention during the FU period. The type of re-intervention is presented in Table 7. 

Five children died after intervention; three of these deaths were attributed to cardiac failure caused by extremely stenotic pulmonary arteries. One had relevant insufficiency after intervention and died after postoperative sepsis. One patient died as a result of complications, including thrombus formation due to an unknown coagulation disorder, following multiple interventions, including cardiac surgery to re-establish a conduit, ECMO application, reduction of ASD and hematoma evacuation, 110 days postpartum. 

**Figure 4:**
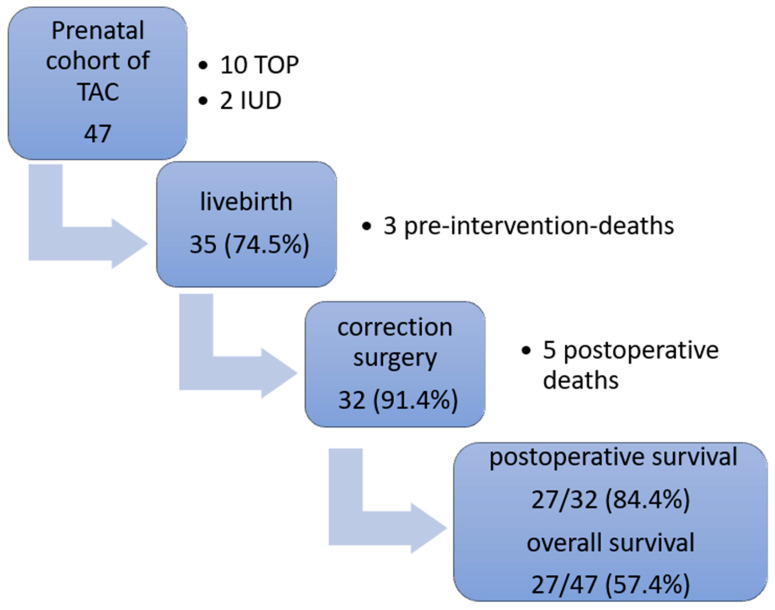
Outcomes within our prenatal TAC cohort. TAC = truncus arteriosus communis; TOP = termination of pregnancy, IUD = intrauterine death.

An overview of the prenatal and postnatal outcomes of our cohort is presented in a flowchart (Figure 4). 

### 3.3. Parameters of Outcome Prediction

In our comparison between the two outcome groups (group 1, comprising subjects who survived throughout the FU period, and group 2, comprising subjects who died during the FU period or in which IUD occurred), no statistically significant differences were observed with respect to any of the parameters. The corresponding *p* values and contingency coefficients are presented in Table 8. 

## 4. Discussion

TAC is a rare congenital heart defect occurring in approximately 1.07 out of every 10,000 live births [2]. Prenatal diagnosis is possible and essential for consulting parents, providing further prenatal examinations such as genetic analysis and enabling the planning of a safe delivery in a specialized center. The available data regarding prenatally diagnosed TAC are limited and comprise very small cohorts, a brief follow-up period or mixed cohorts including both prenatally and postnatally diagnosed cases. The majority of studies are single-center or, on rare occasions, two-center studies.

Data regarding prenatally diagnosed TAC are limited and consist of very small cohorts, short FU period or mixed cohorts including prenatally and postnatally diagnosed cases [7,8,9,12,14,18].

In this study, we evaluated cases from four tertiary centers. To the best of our knowledge, our study represents one of the largest prenatal cohorts to date, comprising more than three centers and a long postnatal follow-up period.

One of the objectives of our evaluation was to examine the prevalence of associated anomalies in fetuses with TAC.

The prevalence of genetic anomalies and extracardiac anomalies in our cohort, at approximately 40% and 36%, respectively, aligns with the reported averages of 30% and 36% in the existing literature, as summarized in a recent review [13]. The most prevalent anomaly observed in our cohort was microdeletion 22q11.2 as previously reported by others [8,10,13].

In their review, Nisselrooij et al. report a rate of 39% for additional intracardiac anomalies [13]. The rate in our cohort is higher, at over 63% when coronary anomalies are excluded and 68.1% when they are included. A comparable high prevalence of associated intracardiac anomalies was observed in a prenatal cohort by Lee et al. Similarly, they considered ASD and RAA as additional intracardiac anomalies [14]. When considering only major cardiac defects, the rate in our cohort was found to be 8.5%, which is similar to the results reported by Abel et al. [8].

Table 9 provides an overview of the associated anomalies and outcomes, which are compared to the results reported in other articles.

To the best of our knowledge, our work represents one of the first reports on the prevalence of coronary anomalies in a prenatal cohort with TAC. The prevalence in our cohort was 17%, which is consistent with reported incidence range of 5–20%. It is possible that the incidence is in fact higher, as some cases may be missed by postnatal preoperative echocardiography and only detected intraoperatively [20].

Rodefeld et al. highlight the significance of coronary artery anatomy, particularly for surgeons. Inadvertent injury can occur during removal of the pulmonary arteries from the truncal root or the closure of the residual defect in the truncal root resulting from the pulmonary artery dissection during repair and the replacement of the truncal valve. Furthermore, it is crucial to be aware of coronary artery variations for administration of cardioplegia, particularly when second doses are directly injected into the coronary ostia and there is a risk of inadequate cardioplegia flow in the event of early bifurcation [21]. Additionally, others have identified coronary anomalies as risk factor for perioperative mortality and late mortality in postnatal life [20,22]. No significant difference was identified in the prevalence of coronary anomalies between survivors and non-survivors within our cohort. Nevertheless, it is recommended that coronary anomalies be considered as a risk factor when counseling parents.

Information regarding the various prenatal TAC subtypes is scarce, with only two research groups reporting on this topic [8,14]. The distribution of TAC subtypes in our cohort is comparable to that observed in postnatal series, with Van Praagh type 1 being the most prevalent, followed by type 2 [23,24]. In contrast to the prenatal cohort of Abel et al., [8].which reported a rate of 33% for subtype A4, we found a relatively low incidence of approximately 10% of subtype 4, as evidenced by pediatric data [23,24].

The neonatal data reveals adverse outcome with a high mortality rate in cases with TAC and IAA (interrupted aortic arch) corresponding to TAC subtype 4 [25].

Nevertheless, our results indicate that TAC subtype 4 does not result in a more unfavorable outcome. This is consistent with the findings of Abel et al., who reported that the outcome was favorable regardless of TAC subtype and that there were no adverse outcomes associated with type 4 [8]. It should be noted that the neonatal data indicating an adverse outcome for TAC subtype 4 are over 25 years old and that the authors noted slight improvement of overall survival even within their study period of 10 years. Otherwise, the postnatal FU period in the aforementioned study was relatively lengthy, spanning 15 years [25], such that that mortality may have been underestimated in our evaluation. According to our results and those of Abel et al., [8]. in a recent evaluation of prenatally diagnosed TAC cases, it can be concluded that IAA alone does not significantly elevate the risk of mortality.

With regard to prenatal outcome in our cohort, nearly one-fifth (21.3%) opted for TOP. This is in accordance with others reporting TOP rates between 17 and 75% [7,9,10,13,14,15,19].

As observed by others [19], most cases in our cohort were non-isolated cases with severe extracardiac and/or chromosomal anomalies, such that the parents’ decision would be influenced by the associated anomalies rather than the cardiac defect itself.

The incidence of IUD in our cohort with 4% was in the lower range compared with literature data, reporting rates between 5 and 13% [8,9,10,13,19].

With regard to postnatal outcomes, 74.5% of our prenatal cohort were born alive. Three (8.6%) patients with non-isolated TAC died prior to surgery, in agreement with reported pre-surgical mortality rates ranging from 3% to almost 31% [8,9,10,12,13,14,19].

Initial intervention was performed at a median of 16 days postpartum. This is consistent with the findings of previous studies, which have reported increasing numbers of surgeries performed during the neonatal period over time, with a median age of 13 days [20]. Some argue for repair as early as possible, ideally within the first day of life, to avoid pulmonary overcirculation and heart failure [21]. Conversely, others found significantly poorer survival rates in neonates who have undergone surgical repair in the first or fourth week of life compared to week 2 or 3. Patients who underwent surgery during the first week in their cohort had a higher incidence of significant comorbidities and were in a more critical state whereas surgery in week 4 resulted in more overcirculation and worsening heart failure [20]. In a cohort with prenatally diagnosed cases who underwent surgery within the first week of life, mortality was higher than in the postnatally diagnosed cases who underwent repair at a median age of 12 days. However, this may also be attributed to a higher prevalence of associated comorbidities, which may have resulted in a greater incidence of complications in the group diagnosed prenatally [9].

The repair operation rate in our cohort was notably high, at 91.4%. During the FU period, numerous re-interventions were observed (68.8%) in accordance with postnatal pediatric data reporting a re-intervention rate of approximately 77% over a 10-year follow-up period. The majority of these re-interventions were right ventricular outflow tract reconstruction followed by truncal valve repair/replacement and some relief of aortic obstruction [26].

Postsurgical FU data in cohorts with prenatal diagnosis are scarce. Abel et al. reported complete cardiac repair in 78% of their prenatal cohort and also observed a high rate of re-interventions, 46.2%, during the FU period [8]. We documented a post-operative survival rate of almost 85% during a median postnatal FU of 51.5 months. All postoperative deaths occurred within the first postoperative months, which corresponds to recent data on long-terms outcome after surgery. These data indicate that over 80% of deaths occur within the first year after surgery [20].

With regard to prenatal predictors, no significant difference was observed between the evaluated parameters of the survivor and non-survivor groups. In postnatal life, truncal dysfunction and regurgitation are considered risk factors for adverse outcome and recurrent surgery [27], but we could not work this out in our prenatal cohort. Some authors also consider truncal regurgitation a prenatal parameter predicting an adverse outcome; however, the majority of cohorts are insufficiently sized for statistical evaluation and the drawing of conclusions [9,10,12]. Otherwise, Lee et al. found no correlation between valve features and postnatal outcomes, but their number of cases was also insufficient for meaningful statistical evaluation [14]. A recent retrospective cohort study could work out ventricular dysfunction, low CVP (cardiovascular profile) score, skin edema and abnormal umbilical venous Doppler as risk parameters for presurgical mortality in a prenatal cohort. They also found a tendency toward worse outcome in fetuses with tricuspid regurgitation and stenosis, but this parameter did not reach significance. However, truncal regurgitation and stenosis may undergo progression during pregnancy, thereby becoming relevant for the development of valve dysfunction [19].

Associated genetic and extracardiac anomalies can be assumed to be a risk factor for pre- and postnatal mortality. Nisselroij et al. confirmed this in their recent analysis demonstrating that genetic or extracardiac anomalies were present in 75% of non-survivors (IUFD and neonatal deaths), compared to only 31% of survivors [13]. This is in contrast to our results and others who did not identify a significant association between pre-surgical or prenatal death and additional fetal anomalies [9,19]. However, this may be subject to bias due to the fact that in non-isolated cases with presumed adverse outcomes, TOP was performed.

With regard to low birth weight and prematurity as risk factors for adverse outcomes, the data is more consistent [13,19,20]. Despite the absence of significant differences in FGR rates between survivors and non-survivors, the majority of non-survivors had a birth weight below 3000 g, and prematurity emerged as the parameter with the greatest tendency towards significance for mortality in our results. This should inform the regarding delivery planning after 37 weeks of gestation in patients with a prenatal diagnosis of TAC, in a manner analogous to the approach taken for CHD in general.

## 5. Conclusions

TAC is a rare congenital heart defect with only limited outcome data from a low number of cases, especially for prenatally diagnosed cases. Prenatal TAC cases are closely associated with additional genetic abnormalities, particularly 22q11.2 microdeletion, and extracardiac anomalies, which occurred in 36% and 40% of cases, respectively, in our cohort. The postoperative survival rate for live births with intention to treat was high, at almost 85%.

However, a considerable number of cases (approximately two-thirds) required re-interventions during the follow-up period. With regard to prenatal predictors, no significant difference was observed between the survivor and non-survivor groups with respect to the evaluated parameters. To the best of our knowledge, this is the largest prenatal cohort with one of the longest FU periods and the only one to have been recruited from more than three centers.

## Figures and Tables

**Table 1:** Subtypes of TAC in the Van Praagh classification [5].

	Each type may include a modifier: “A” (with VSD) or “B” (intact ventricular septum).
subtype A1	The main pulmonary artery is present and bifurcates into the left and right pulmonary arteries
subtype A2	The right and left branch pulmonary arteries arise from the common trunk without a main pulmonary trunk
subtype A3	One branch of the pulmonary artery (typically the right) arises from the common trunk, and the other arises from a ductus arteriosus or the aorta
subtype A4	Presence of aortic arch hypoplasia, coarctation or interrupted aortic arch and a large ductus arteriosus.

**Table 2:** TAC subtype and common trunk valve details in our prenatal cohort with TAC.

prenatal classification into Van Praagh subtype possible	37/47 (78.7%)
type A 1	18/37 (48.6%)
type A 2	13/37 (35.1%)
type A 3	2/37 (5.4%)
type A 4	4/37 (10.8%)
existing data about common trunk valve details	33/47 (70.2%)
unicuspid valve	1/33 (3.0%)
bicuspid valve	7/33 (21.2%)
tricuspid valve	13/33 (39.4%)
quadricuspid valve	12/33 (36.4%)
valve insufficiency	14/47 (29.8%)
valve stenosis	8/47 (17.0%)

**Table 3:** All cases with additional extracardiac anomalies within our cohort.

Case	Chromosomal Anomaly	Additional Cardiac Malformation	Additional Extracardiac Malformation	Outcome
1	22q11.2 microdeletion (del 22q11.2)	ASD + RAA	thymic aplasia (22q11.2 microdeletion)	alive
2	del 22q11.2	ASD	thymic aplasia 34	alive
3	-	SCA	anal atresia	alive
4	n.a.	RAA	esophageal atresia, biliary atresia and renal hypoplasia	died within 7 days postpartum
5	n.a.	ASD, RAA, high take-off of the RCA	spondylocostal dysostosis	alive
6	n.a.	-	thymic aplasia	alive
7	n.a.	-	thymic aplasia	alive
8	n.a.	-	thymic aplasia	alive
9	-	ASD+ RAA	microcephaly	alive
10	del 22q11.2	ASD	thymic aplasia, clubfeet	alive
11	-	-	SUA + ductus venosus agenesis	IUD
12	trisomy 13	ASD	central nervous system anomaly, spina bifida, hydronephrosis, hexadactyly	TOP
13	trisomy 13	-	clubfeet, hexadactyly (trisomy 13)	TOP
14	trisomy 13	AVSD	SUA, spina bifida, hexadactyly, microcephaly, cleft lip and palate (trisomy 13)	TOP
15	n.a.	ASD	thymic aplasia, hexadactyly, renal agenesis	TOP
16	n.a.	hypoplastic heart	anencephaly	TOP
17	-	ASD	SUA, hydronephrosis, renal agenesis, hemivertebra, plantar cutaneous appendage	died

ASD = atrial septal defect; RAA = right aortic arch; SCA = single coronary artery; RCA = right coronary artery; SUA = single umbilical artery; IUD = intrauterine death; TOP = termination of pregnancy, n.a. = not assessed.

**Table 4:** All cases with additional intracardiac anomalies but without extracardiac anomalies.

Case	Chromosomal Anomaly	Additional Cardiac Malformation	Additional Extracardiac Malformation	Outcome
1	46, X,dup (y) (q12) {10}	LPSVC + ASD, high take-off of the right coronary artery	-	alive
2	n.a.	RAA + ASD, high take-off of the right coronary artery (RCA)	-	alive
3	del 22q11.2	RAA	-	alive
4	n.a.	RAA	-	alive
5	del 22q11.2	multiple VSD, RAA	-	alive
6	del 22q11.2	RAA	-	alive
7	del 22q11.2	ASD	-	alive
8	-	ASD, SCA	-	alive
9	n.a.	LPSVC, high take-off of the RCA and intramural LCA from RCA	-	died
10	-	RAA, high take-off of the RCA and intramural RCA	-	alive
11	-	anomalous origin of LCA	-	died

LPSVC = left persistent superior vena cava; ASD = atrial septal defect; RAA = right aortic arch; RCA = right coronary artery; VSD = ventricular septal defect; SCA = single coronary artery; LCA = left coronary artery, n.a. = not assessed.

**Table 5:** Additional chromosomal anomalies in members of our cohort without further extracardiac/intracardiac anomalies.

Case	Chromosomal Anomaly	Additional Cardiac Malformation	Additional Extracardiac Malformation	Outcome
1	del 22q11.2	-	-	died
2	T13	-	-	TOP
3	T18	-	-	TOP
4	del 22q11.2	-	-	TOP

TOP = termination of pregnancy.

**Table 6:** Isolated cases without extracardiac, further intracardiac anomaly (including anomalies of coronary arteries) or genetic anomaly.

Case	TAC- Type	Chromosomal Anomaly	Additional Cardiac Malformation	Additional Extracardiac Malformation	Outcome
1	1	-	-	-	alive
2	1	-	-	-	alive
3	1	-	-	-	alive
4	2	-	-	-	IUD
5	unknown	-	-	-	TOP
6	1	-	-	-	TOP

IUD = intrauterine death; TOP = termination of pregnancy.

**Table 7:** Re-intervention in 22 patients in our cohort.

Case 1	conduit re-operation
Case 2	conduit re-operation + aortic valvuloplasty
Case 3	conduit re-operation+ repeat atrioventricular valvuloplasty
Case 4	conduit re-operation
Case 5	conduit re-operation + aortic valvuloplasty
Case 6	conduit re-operation + RPA angioplasty
Case 7	conduit re-operation + aortic valvuloplasty
Case 8	conduit re-operation + conduit balloon angioplasty
Case 9	conduit re-operation
Case 10	conduit and LPA balloon angioplasty
Case 11	conduit re-operation + LPA & RPA angioplasty + conduit balloon angioplasty and pulmonary artery (PA) balloon angioplasty
Case 12	conduit re-operation + aortic valvuloplasty + RPA & LPA balloon angioplasty
Case 13	PA angioplasty
Case 14	conduit re-operation
Case 15	conduit re-operation + aortic valvuloplasty
Case 16	conduit and LPA & RPA balloon angioplasty
Case 17	LPA balloon angioplasty
Case 18	conduit re-operation + PA angioplasty
Case 19	PA balloon angioplasty
Case 20	conduit re-operation + PA balloon angioplasty
Case 21	conduit re-operation
Case 22	conduit re-operation, ASD reduction, various thorax openings and closings because of hematoma and ECMO

RPA = right pulmonary artery; LPA = left pulmonary artery; PA = pulmonary artery; ASD = atrial septal defect; ECMO = extracorporeal membrane oxygenation.

**Table 8:** Comparison of different parameters between the two outcome groups (group 1: survivor; group 2: non-survivor (IUD/postnatal death).

Comparison Parameter	Group 1 (Survivor)	Group 2 (Non-Survivor)	*p* Value	Cramer’s V
TAC subtype	14 (subtype 1)	4 (subtype 1)	1.0	0.0645
	9 (subtype 2)	4 (subtype 2)	0.8415	0.1
	1 (subtype 3)	0 (subtype 3)	0.5598	0.0972
	3 (subtype 4)	1 (subtype 4)	0.5376	0
		1 × unknown (IUD patient)		
number of valve leaflets	1 unicuspid	0 unicuspid	0.4028	0.0835
	6 bicuspid	1 bicuspid	0.8065	0.0522
	9 tricuspid	4 tricuspid	0.2943	0.2629
	11 quadricuspid	1 quadricuspid	0.522	0.1931
		4 unknown		
valve stenosis	5/27	3/10	0.7642	0.1241
valve insufficiency	12/27	2/10	0.3272	0.2236
presence of genetic anomaly	8/27	1/10	0.4201	0.2034
presence of extracardiac anomaly	9/27	3/10	0.8415	0.0329
presence of FGR	4/27	3/10	0.5657	0.1724
presence of additional intracardiac anomaly	21/27	7/10	1.0	0.0805
ASD	14	3	0.4166	0.1945
RAA	11	2	0.431	0.1931
cardiomegaly	1	0	0.5967	0.1013
prematurity	1/27	3/10	0.0908	0.376

TAC = truncus arteriosus communis; FGR = fetal growth restriction, ASD = atrial septal defect; RAA = right aortic arch.

**Table 9:** An overview of associated anomalies and outcome compared to results by other articles.

Autor	Our Results	Cox et al., 2023 [19] *Pediatr Cardiol.*	Van Nisselroij et al., 2021 [13] *Prenatal Diagnosis*	Abel et al., 2021 [8] *Archives of Gynecology and Obstetrics*	Mărginean et al., 2018 [15] *Medical Ultrasono-graphy*	Gomez et al., 2016 [7] *Fetal Diagn Ther*	Lee et al., 2013 [14] *Fetal Diagn Ther*	Swanson et al., 2009 [9] *Pediatr Cardiol.*	Volpe et al., 2003 [10] *Heart*	**Duke et al.,** **2001** [12] ***Am J Cardiol.***
recruitment	2008–2021	2010–2020	2002–2016	2010–2018	2009–2017	2006–2015	2003–2012	1992–2007	1993–2002	1990–1999
cases	47	23	38	34 confirmed cases	17	10	12	43 prenatal cases	23	17
centers	4	2	1	2	1	1	1	1	3	1
diagnosis (pre-/postnatal)	pre	pre	pre	pre	pre	pre	pre	pre + post	pre	pre
TOP	10/47 = 21.3%	4/23 = 17.4%	18/38 = 47.3%	14/34 = 41.2%	8/17 = 47.0%	9/10 = 90.0% of confirmed TAC	4/12 = 33.3%	17/43 = 39.5%	8/23 = 34.8%	4/17 = 23.5%
IUD	2/47 = 4.3%	2/19 = 10.5%	2/38 = 5.3%	1/34 = 2.9%	-	-	-	2/43 = 4.7%	2/23 = 8.7%	-
live births	35/47 = 74.5%	17/23 = 73.9%	18/38 = 47.3%	19/34 = 55.8%	4/17 = 23.5%	1/10 = 10.0%	8/12 = 66%	24/43 = 55.8% 19/43 = 44.1% with confirmed TAC	13/23 = 56.5%	13/17 = 76.5%
overall survival	27/47 = 57.4%	14/23 = 93.3%	12/38 = 31.6%	14/34 = 41.2%	1/17 = 5.9%	1/10 = 10.0%	6/12 = 50.0%	13/43 = 30.2% 13/38= 34.2% when considered postnatal confirmed TAC)	8/23 = 34.8%	5/17 = 29.4%
intention-to-treat survival	27/32 = 84.4% 3 pre surgical deaths in non-isolated cases	14/17 = 82.4% 2 pre-surgical deaths	12/16 = 75.0% 2/18 prenatal death (without active treatment) and 2 other pre-surgical deaths with ITT)	14/19 = 73.7%	1/3 = 33.3%	1/1 = 100%	6/7 = 85.7% 1 presurgical death in non-isolated form)	13/19 = 68.4% of confirmed TAC cases	8/10 = 80.0% 3 pre-surgical deaths)	5/8 = 62.5% 4 presurgical deaths
peri-/postoperative mortality	5/32 = 15.6%	1/15 = 0.7%	2/14 = 14.3%	5/19 = 26.3%	2/3 = 66.7%	0	1/7 = 14.3%	4/17 = 23.5%	2/8 = 25.0%	3/8 = 37.5% but one palliative operation 2/7 = 28.6% with repair operation
associated anomalies (genetic)	15/47 = 34.1% of prenatal cases 15/37 = 40.5% with invasive diagnostics	1/23 = 47.8% of prenatal cases 11/23 = 47.8% with invasive diagnostics	15/38 = 39.5% of prenatal cases 15/38 = 39.5% with invasive diagnostics	13/34 = 38.2% of prenatal cases 13/25 = 52.0% with invasive diagnostics	1/17 = 5.9% of prenatal cases 1/5 = 20.0% with invasive diagnostics	4/10= 40.0% of prenatal cases 4/10= 40.0% with invasive diagnostics	2/12 = 16.7% of prenatal cases 2/12 = 16.7% with invasive diagnostics	5/43 = 11.6% of prenatal cases 5/17 = 29.4% with invasive diagnostics	8/23 = 34.8% of prenatal cases 8/22 = 36.4% with invasive diagnostics	3/17 = 17.6% of prenatal cases 3/10 = 30.0% with invasive diagnostics for microdel
microdel 22q11.2	9 /37 = 24.3% with invasive diagnostics	4/21 = 19.0% with invasive diagnostics	8/38 = 21.1% with invasive diagnostics	6/25 = 24.0% with invasive diagnostics	0/5 = 0% with invasive diagnostics	1/10 = 10.0% of confirmed cases with invasive diagnostics	0/9 = 0% with invasive diagnostics for microdel	5/17 = 29.4% with invasive diagnostics	6/19 = 31.6% with invasive diagnostics for microdel	3/10 = 30.0% with invasive diagnostics for microdel
extra- cardiac anomalies	17/47 = 36.2%	10/23 = 43.5%	20/38 = 52.6%	20/34 = 58.8%	5/17 = 29.4%	4/10 = 40.0%	2/12 = 16.7%	6/19 = 31.6%	10/23 = 43.5%	4/17 = 23.5%
additional intra-cardiac anomalies	30/47 = 63.8% excluding coronary anomalies, 32/47 = 68.1% including coronary anomalies 4/47 = 8.5% “major” cardiac anomalies	not reported	14/38 = 36.8%	15/34 = 44.1% including minor defects 3/34 = 8.8% “major” cardiac anomalies	not reported	2/10 = 20.0%	9/12 = 75.0%	not reported	8/23 = 34.8%	not reported
surgical repair	32	15	14	13 done, 1 planned	3 (1 of them only first step)	1	7 (+1 intraoperative death)	17 (of prenatal cases)	6 (+2 palliative and +2 awaiting surgery)	7 (+1 palliative)
FU (months)	52 (median)	not reported (FU until discharge from hospital)	72 (mean)	42 (mean)	4 and 8 months (both died), one alive 2 months	10 (only one patient)	38.5 (mean) 41 (median)	not reported	10 (median)	41 (median)

TOP = termination of pregnancy, IUD = intrauterine death; TAC = truncus arteriosus communis; ITT = intention-to-treat; microdel = microdeletion; FU = follow-up.

## Data Availability

The data that support the findings of this study are available from the corresponding author upon reasonable request.

## References

[1] Webb S., Qayyum S.R., Anderson R.H., Lamers W.H., Richardson M.K. (2003). Septation and separation within the outflow tract of the developing heart. J. Anat..

[2] Hoffman J.I.E., Kaplan S. (2002). The incidence of congenital heart disease. J. Am. Coll. Cardiol..

[3] Jacobs M.L. (2000). Congenital Heart Surgery Nomenclature and Database Project: Truncus arteriosus. Ann. Thorac. Surg..

[4] Van Praagh R., Van Praagh S. (1965). The anatomy of common aorticopulmonary trunk (truncus arteriosus communis) and its embryologic implications. A study of 57 necropsy cases. Am. J. Cardiol..

[5] Van Praagh R. (1976). Classification of Truncus Arteriosus Communis. Am. Heart J..

[6] Patel A., Costello J., Backer C., Pasqualii S., Hill K., Wallace A., Jacob J., Jacobs M. (2016). Prevalence of Noncardiac and Genetic Abnormalities in Neonates Undergoing Cardiac Operations: Analysis of The Society of Thoracic Surgeons Congenital Heart Surgery Database. Ann. Thorac. Surg..

[7] Gómez O., Soveral I., Bennasar M., Crispi F., Masoller N., Marimon E., Bartrons J., Gratacós E., Martinez J.M. (2016). Accuracy of Fetal Echocardiography in the Differential Diagnosis between Truncus Arteriosus and Pulmonary Atresia with Ventricular Septal Defect. Fetal Diagn. Ther..

[8] Abel J.S., Berg C., Geipel A., Gembruch U., Herberg U., Breuer J., Brockmeier K., Gottschalk I. (2021). Prenatal diagnosis, associated findings and postnatal outcome of fetuses with truncus arteriosus communis (TAC). Arch. Gynecol. Obstet..

[9] Swanson T., Tierney E., Tworetzky W., Pigula F., McElhinney D. (2009). Truncus Arteriosus: Diagnostic Accuracy, Outcomes, and Impact of Prenatal Diagnosis. Pediatr. Cardiol..

[10] Volpe P., Paladini D., Marasini M., Buonadonna A.L., Russo M.G., Caruso G., Marzullo A., Vassallo M., Martinelli P., Gentile M. (2003). Common arterial trunk in the fetus: Characteristics, associations, and outcome in a multicentre series of 23 cases. Heart.

[11] Gul A., Corbacioglu A., Bakirci I.T., Ceylan Y. (2012). Associated anomalies and outcome of fetal aberrant right subclavian artery. Arch. Gynecol. Obstet..

[12] Duke C., Sharland G., Jones A., Simpson J. (2001). Echocardiographic features and outcome of truncus arteriosus diagnosed during fetal life. Am. J. Cardiol..

[13] van Nisselrooij A.E.L., Herling L., Clur S.A., Linskens I.H., Pajkrt E., Rammeloo L.A., ten Harkel A.D.J., Hazekamp M.G., Blom N.A., Haak M.C. (2021). The prognosis of common arterial trunk from a fetal perspective: A prenatal cohort study and systematic literature review. Prenat. Diagn..

[14] Lee M.-Y., Won H.-S., Lee B.S., Kim E.A.-R., Kim Y.-H., Park J.-J., Yun T.-J. (2013). Prenatal diagnosis of common arterial trunk: A single-center’s experience. Fetal Diagn. Ther..

[15] Mărginean C., Gozar L., Mărginean C.O., Suciu H., Togănel R., Muntean I., Mureşan M.C. (2018). Prenatal diagnosis of the fetal common arterial trunk. A case series. Med. Ultrason..

[16] Thompson L., McElhinney D., Reddy M., Petrossian E., Silverman N., Hanley F. (2001). Neonatal repair of truncus arteriosus: Continuing improvement in outcomes. Ann. Thorac. Surg..

[17] Carvalho J.S., Allan L.D., Chaoui R., Copel J.A., DeVore G., Hecher K., Lee W., Munoz H., Paladini D., Tutschek B. (2013). ISUOG Practice Guidelines (updated): Sonographic screening examination of the fetal heart. Ultrasound Obs. Gynecol..

[18] McElhinney D.B., Clark B.J., Weinberg P.M., Kenton M.L., McDonald-McGinn D., Driscoll D.A., Zackai E.H., Goldmuntz E. (2001). Association of chromosome 22q11 deletion with isolated anomalies of aortic arch laterality and branching. J. Am. Coll. Cardiol..

[19] Cox K., Husain N., Jhaveri S., Geiger M., Berhane H., Patel S. (2023). Fetal Echocardiographic Variables Associated with Pre-Surgical Mortality in Truncus Arteriosus: A Pilot Study. Pediatr. Cardiol..

[20] Naimo P.S., Bell D., Fricke T.A., d’Udekem Y., Brizard C.P., Alphonso N., Konstantinov I.E. (2021). Truncus arteriosus repair: A 40-year multicenter perspective. J. Thorac. Cardiovasc. Surg..

[21] Rodefeld M.D., Hanley F.L. (2002). Neonatal truncus arteriosus repair: Surgical techniques and clinical management. Pediatr. Card. Surg. Annu..

[22] Hanley F., Heinemann M., Jonas R., Mayer J.J., Cook N., Wessel D., Castaneda A. (1993). Repair of truncus arteriosus in the neonate. J. Thorac. Cardiovasc. Surg..

[23] Calder L., Van Praagh R., Van Praagh S., Sears W.P., Corwin R., Levy A., Keith J.D., Paul M.H. (1976). Truncus arteriosus communis. Clinical, angiocardiographic, and pathologic findings in 100 patients. Am. Heart J..

[24] Miyamoto T., Sinzobahamvya N., Kumpikaite D., Asfour B., Photiadis J., Brecher A.M., Urban A.E. (2005). Repair of truncus arteriosus and aortic arch interruption: Outcome analysis. Ann. Thorac. Surg..

[25] Konstantinov I.E., Karamlou T., Blackstone E.H., Mosca R.S., Lofland G.K., Caldarone C.A., Williams W.G., Mackie A.S., McCrindle B.W. (2006). Truncus arteriosus associated with interrupted aortic arch in 50 neonates: A congenital heart surgeons society study. Ann. Thorac. Surg..

[26] Alfieris G.M., Swartz M.F. (2016). The Initial Glimpse at Long-term Outcomes Following the Repair of Truncus Arteriosus. Semin. Thorac. Cardiovasc. Surg..

[27] Parikh R., Eisses M., Latham G., Joffe D., Ross F. (2018). Perioperative and Anesthetic Considerations in Truncus Arteriosus. Semin. Cardiothorac. Vasc. Anesth..

